# Time Series Prediction of Open Quantum System Dynamics by Transformer Neural Networks

**DOI:** 10.3390/e28020133

**Published:** 2026-01-23

**Authors:** Zhao-Wei Wang, Lian-Ao Wu, Zhao-Ming Wang

**Affiliations:** 1 College of Physics and Optoelectronic Engineering, Ocean University of China, Qingdao 266100, China; wangzw_ouc@163.com; 2 Department of Physics, University of the Basque Country UPV/EHU, 48080 Bilbao, Spain; lianao_wu@ehu.es; 3 IKERBASQUE, Basque Foundation for Science, 48013 Bilbao, Spain; 4 EHU Quantum Center, University of the Basque Country UPV/EHU, 48940 Leioa, Spain; 5 Engineering Research Center of Advanced Marine Physical Instruments and Equipment of Ministry of Education, Ocean University of China, Qingdao 266100, China; 6 Qingdao Key Laboratory of Optics and Optoelectronics, College of Physics and Optoelectronic Engineering, Ocean University of China, Qingdao 266100, China

**Keywords:** time series prediction, open quantum system, machine learning, Transformer neural networks

## Abstract

The dynamics of open quantum systems play a crucial role in quantum information science. However, obtaining numerically exact solutions for the Lindblad master equation is often computationally expensive. Recently, machine learning techniques have gained considerable attention for simulating open quantum system dynamics. In this paper, we propose a deep learning model based on time series prediction (TSP) to forecast the dynamical evolution of open quantum systems. We employ the positive operator-valued measure (POVM) approach to convert the density matrix of the system into a probability distribution and construct a TSP model based on Transformer neural networks. This model effectively captures the historical evolution patterns of the system and accurately predicts its future behavior. Our results show that the model achieves high-fidelity predictions of the system’s evolution trajectory in both short- and long-term scenarios, and exhibits robust generalization under varying initial states and coupling strengths. Moreover, we successfully predicted the steady-state behavior of the system, further proving the practicality and scalability of the method.

## 1. Introduction

The dynamics of open quantum systems represent a fundamental subject for understanding microscopic mechanisms—including dissipation, decoherence, and information loss [1]. Moreover, they constitute a core prerequisite for realizing practical quantum technologies, from quantum communication [2] and computing [3,4] to quantum information processing [5,6]. To simulate the reduced dynamics derived from the full system-environment Hamiltonian, a range of numerically exact methods have been developed. Notable examples include the hierarchy of equations of motion [7], path integral Monte Carlo [8], the time-evolving matrix product operator method [9], and quantum state diffusion [10,11,12]. Despite their precision, the computational cost of these algorithms typically scales exponentially with both the system size and the number of evolution steps, making them prohibitively expensive for simulating long-time quantum dynamics.

Recently, machine learning methods have been increasingly applied to study open quantum system dynamics [13,14,15]. One common strategy treats the system’s evolution as an optimizable path, parameterized by neural networks for their strong representational capacity, and identifies the optimal trajectory via variational principles [16,17,18]. For instance, Reh et al. [16] developed a time-dependent variational principle in the positive operator-valued measure (POVM) representation and implemented local explicit parameter updates using autoregressive neural networks, enabling efficient simulation of one- and two-dimensional open quantum many-body systems. In a similar vein, Luo et al. [17] parameterized quantum state distributions via autoregressive neural networks and incorporated string states to enhance symmetry, achieving high-precision simulations of both dynamical and steady-state behaviors in open quantum systems. Another line of research employs physics-informed neural networks (PINNs) to directly solve the underlying differential equations governing system dynamics [19,20,21]. For example, Norambuena et al. [19] embedded the Lindblad master equation into the loss function as a physical constraint, enabling the design of smooth control fields for high-fidelity state transfer in open quantum systems. Ullah et al. [22] developed a new method that integrates PINNs with uncertainty-aware hard constraints, ensuring strict physical conservation laws (such as trace conservation) in the simulation of quantum dissipative dynamics by design. This addresses the issue of physical inconsistencies that may arise from purely data-driven neural networks. Both variational and PINN-based approaches have achieved notable success. However, they also exhibit inherent limitations. Variational methods often involve complex optimization procedures that are difficult to stabilize and are highly sensitive to network architecture and hyperparameters, hindering their reliable deployment in practical settings. On the other hand, PINNs strongly depend on an exact mathematical description of the system dynamics. When the model is imperfect or affected by uncharacterized noise, their control accuracy and generalization capability can degrade significantly.

Beyond the above paradigms, an alternative approach involves learning intrinsic dynamical mappings directly from high-quality data [23,24]. This strategy does not rely explicitly on specific physical equations, offering greater robustness against model inaccuracies. Moreover, once trained, such models enable extremely fast forward inference, allowing nearly real-time prediction and control—an essential feature for applications such as quantum feedback control. Time series prediction (TSP), in particular, uses machine learning to analyze short-term evolution data in order to forecast long-term dynamics, thereby circumventing high computational costs [25,26,27]. Since early-stage evolution often encodes information about future dynamics, it is possible to predict long-term behavior from short-time trajectories [28]. Various neural architectures—including Convolutional Neural Networks [29], Long Short-Term Memory networks [30], and hybrid models [31]—have been used to predict the population and coherence dynamics of two-level systems in open environments. Among them, Transformer neural networks [32], with their self-attention mechanisms and capacity for capturing long-range dependencies, are naturally suited to model global correlations in non-unitary open system dynamics. Rodríguez and Kananenka [33] demonstrated that a Transformer-based model trained on short trajectories can accurately predict the long-time population dynamics of open quantum systems in dissipative environments. These works mostly use specific physical quantities such as $\langle {\hat{\sigma }}_{z}\rangle$ as time series, which is not sufficient in fully describing the dynamical evolution of open systems. The density matrix ρ can fully describe the information of a quantum system. Unless it describes a pure state system, the density matrix is always a complex matrix, with its coherences always being complex-valued, making it difficult to directly use established machine learning tools for processing.

In this work, we convert the density matrix ρ into a one-dimensional probability distribution real vector using the POVM method and develop a TSP model based on Transformer neural networks to simulate the evolution of the density matrix ρ of open quantum systems. We evaluate the performance of the model by direct comparison with numerical reference solutions. Our results indicate that the model effectively learns the dynamical evolution, yielding accurate predictions over both short and long timescales for various initial states and coupling strengths. Additionally, the model demonstrates the capability to predict the system’s steady state. These findings suggest that the presented approach offers a useful and efficient alternative for simulating open quantum dynamics.

## 2. Methods

Born–Markovian approximation is commonly used to describe the open system; in this case, the Lindblad master equation takes the form

(1) $$\dot{\rho }=-i\left[H,\rho \right]+\sum_{k}\frac{\gamma_{k}}{2}\left(2L_{k}\rho L_{k}^{†}-\left\{\rho ,L_{k}^{†}L_{k}\right\}\right),$$

where *H* is the system Hamiltonian and ρ is the reduced density matrix of the system, which follows the requirements of the probability conservation and complete positivity of the dynamical map [34]. $\gamma_{k}$ is the decay rate associated with the Lindblad jump operator $L_{k}$.

In order to use the mature machine learning technique, we first apply the POVM to transform the reduced density matrix ρ into one-dimensional probability distribution P(a) [35], where $a=a_{1}\ldots a_{N}$ represents the string of possible measurement outcomes acting on different qubits. Given an information-complete POVM, the probability distribution P(a) can be uniquely mapped to the reduced density matrix ρ of the N-qubit system:

(2) P(a)=tr[ρM(a)],

where $M\left(a\right)=M\left(a_{1}\right)⊗\ldots ⊗M\left(a_{N}\right)$ is one of the *N*-qubit positive semidefinite operators in the set {M(a)}. M(a) is one of the single-qubit measurement bases $\left\{M^{\left(i\right)}\right\}$, which satisfy $\sum_{i}M^{\left(i\right)}=I$ and $M^{\left(i\right)}\geq 0$. In this paper, we use the Tetrahedral POVM $M_{\mathrm{tetra}}={\left\{M^{\left(i\right)}=\left(1/4\right)\left(I+n^{\left(i\right)}\cdot \sigma \right)\right\}}_{i\in \left\{0,1,2,3\right\}}$, whose 4 measurement bases form a regular tetrahedron on the Bloch sphere. The four vectors $n^{\left(i\right)}$ are $n^{\left(0\right)}=\left(0,0,1\right)$, $n^{\left(1\right)}=\left(2\sqrt{2}/3,0,-1/3\right)$, $n^{\left(2\right)}=\left(-\sqrt{2}/3,-\sqrt{6}/3,-1/3\right)$, and $n^{\left(3\right)}=\left(-\sqrt{2}/3,-\sqrt{6}/3,-1/3\right)$. The Tetrahedral POVM has symmetry and information completeness, making it an ideal probabilistic representation. It encodes any single-qubit state into a one-dimensional probability vector, with this linear mapping preserving the learnable structure of quantum dynamics. Its symmetry ensures unbiased features and stable training. This representation also avoids the degenerate boundary problems of probability distributions, thereby significantly enhancing the model’s efficiency and generalization ability in learning evolution patterns from limited data [36].

Equation (2) provides a tensor-product form of the Tetrahedral POVM that can be extended to multi-qubit systems. For example, the measurement basis for a three-qubit system is $a=[a_{1}^{\left(0\right)},a_{2}^{\left(3\right)},a_{3}^{\left(1\right)}]$, which indicates that after measurement, the first, second, and third qubits are in the directions of $n^{\left(0\right)}$, $n^{\left(3\right)}$, and $n^{\left(1\right)}$, respectively. P(a) represents the probability of obtaining this measurement outcome, while M(a) represents the measurement operator associated with this specific measurement result. By inverting Equation (2), we can reconstruct the density matrix:

(3) $$\rho =\sum_{a}\sum_{a^{\prime }}P\left(a\right)T_{{\mathrm{aa}}^{\prime }}^{-1}M\left(a^{\prime }\right),$$

where $T_{{\mathrm{aa}}^{\prime }}=\mathrm{tr}\left(M\left(a\right)M\left(a^{\prime }\right)\right)$ is the element of the overlap matrix *T*. In the probability distribution frame, the expectation value of the operator *O* can be represented by

(4) $$\langle O\rangle =\sum_{a}\sum_{a^{\prime }}P\left(a\right)\mathrm{Tr}\left[OM\left(a^{\prime }\right)T_{a^{\prime }a}^{-1}\right].$$

## 3. TSP and Transformer Neural Networks

**Figure 1:**
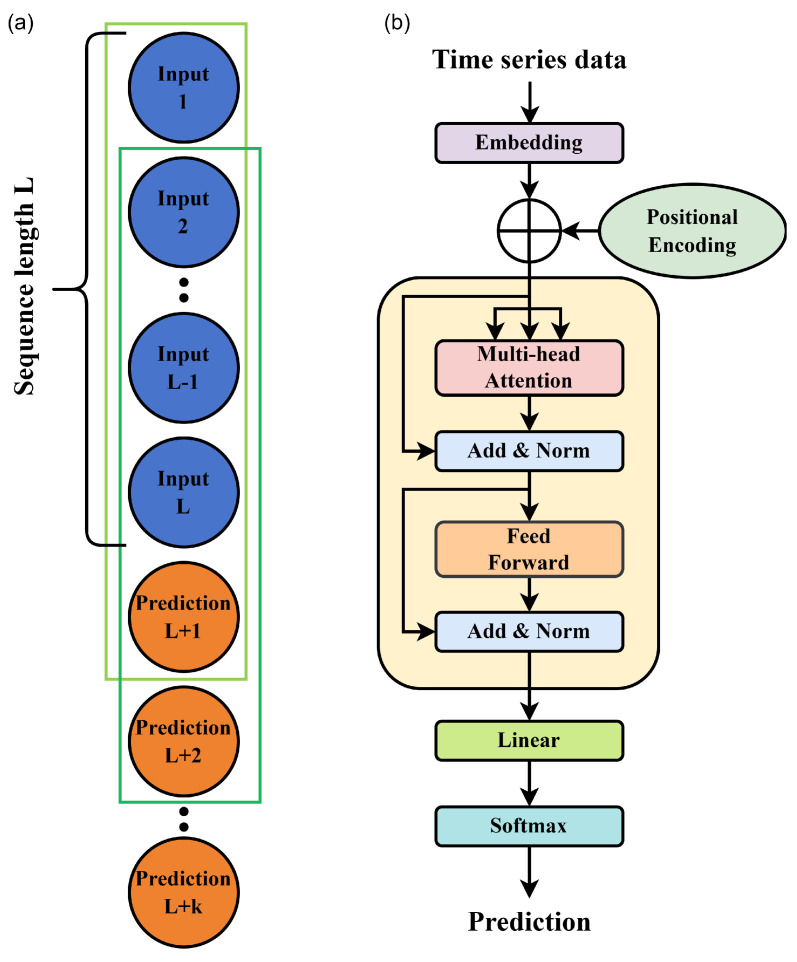
(**a**) Schematic of the TSP. The blue (orange) ball represents the known (predicted) data. A rectangle represents a TSP unit, and *L* is the length of the series. The task is to predict the future (L+1)th data from the *L* known data. (**b**) Schematic of the Transformer model. The time series data is extended by adding Positional Encoding to the Embedding layer. After the data is processed by the Transformer model, the output is obtained through the Linear layer and Softmax layer.

TSP is a method of forecasting the future values based on the characteristics and trends observed in the past time series data. It utilizes statistics and machine learning techniques to build predictive models. Figure 1a presents a schematic of TSP. The rectangular box represents a prediction action unit, where the time series data with a sequence length *L* serves as the basis for the prediction, and the L+1 data point represents the result predicted by the model. To construct a new prediction basis, the L+1 data point is added to the end of the time series, and the first data point is eliminated. This process can be repeated to forecast data for a specific time period in the future. However, it is important to note that this process cannot continue indefinitely. As the forecasting process progresses, an increasing number of predicted data points are utilized to build the forecasting basis. Since the predicted data inherently contains errors compared to the real data, the errors in the later predicted data points will also accumulate.

Our deep learning model utilizes the Transformer neural network in PyTorch 2.0.4 [37] to build an efficient architecture for modeling sequence data. The Transformer model introduces the self-attention mechanism and location coding [32] to enable effective representation and modeling of the input time series data. Figure 1b illustrates a schematic diagram of the Transformer model. In order to be able to input the probability distribution information of multiple moments at once, we arrange the probability distribution of *L* different moments into an Input Matrix. Each row of the Input Matrix represents the probability distribution of measurements at the same time, and each column represents the measurement results of a specific measurement basis at different times. The Embedding layer can make a linear transformation of the time series matrix dimension *d* and extend to the hidden dimension $d_{model}$. Positional Encoding can be computed by the fixed equations (Equations (5) and (6) [32]):

(5) $$PE_{p,i}=\sin\left(p/{10,000}^{2i/d_{model}}\right),$$

(6) $$PE_{p,i}=\cos\left(p/{10,000}^{2i/d_{model}}\right).$$

Since the value does not change, the Positional Encoding matrix can be simply added to the time series matrix at time *t* if there is any data available. *p* represents the number of the time series data bars and *i* represents the number of hidden dimensions $d_{model}$. For odd (even) *i*, the Positional Encoding can be calculated by Equations (5) and (6). The computation at the Encoder layer is parallel, and adding positional coding preserves the time relationship of the time series.

The main principle of the self-attention mechanism is to calculate the attention score of the first moment and every moment in the time series (including the first moment), then multiply the calculated attention score by the information of the corresponding moment, and then add together. The result is the weighted sum of the first moment and all the moments in the time series. Finally, the attention information of each moment and time series is updated in turn. More precisely, we multiply the matrix *A* after Embedding and Positional Encoding by $W^{Q}$, $W^{K}$, and $W^{V}$, respectively, to obtain a query matrix *Q*, a key matrix *K*, and value matrix *V*. Then, the attention value is calculated by Equation (7) [32]:

(7) $$B\left(Q,K,V\right)=\mathrm{softmax}\left(\frac{QK^{T}}{\sqrt{d}}\right)V,$$

where $\mathrm{softmax}\left(x_{i}\right)=e^{x_{i}}/\sum_{j}e^{x_{j}}$. The difference between Multi-Head Attention and Single-Head Attention is that the original three large matrices *Q*, *K*, and *V* are divided into eight small matrices with the same shape (split in the feature dimension), that is, Eight-Head Attention. The results of each small matrix calculation are then spliced together to obtain the same matrix *B* as the results of the Single-Head Attention calculation.

The Add layer is based on the concept of a residual neural network, where the Input Matrix *A* of the Multi-Head Attention is added directly to the output matrix *B* of the Multi-Head Attention, resulting in the sum matrix $B^{\prime }$. Subsequently, Layer Normalization is applied, which normalizes each row of $B^{\prime }$ to follow a standard normal distribution, yielding the final result $B^{\prime \prime }$. The feedforward layer consists of two fully connected layers, with a ReLU activation function [38] sandwiched between them. Finally, the output results are obtained by passing the data through a Linear layer followed by a Softmax activation layer. During training, we employed an Encoder layer and Decoder layer and extended the data to $d_{model}=32$. We employ the Mean Squared Error loss, and the model parameters are updated by the Adam optimizer [39] with default weight initialization and a learning rate of $10^{-5}$. The detailed training process is provided in Appendix A, and the hyperparameters are described in Table A1.

## 4. Results and Discussions

### 4.1. Short-Term TSP

We first analyze the short-term prediction of the TSP model. We consider using TSP to predict the dynamical evolution of a dissipative model with Hamiltonian $H=g\sigma_{z}$ and Lindblad operator $L_{k}=\sigma_{-}=\frac{1}{2}\left(\sigma_{x}-i\sigma_{y}\right)$, where g=−0.5π (the Planck constant *ℏ* is set to 1). The length of the time series *L* will have a direct impact on the model’s prediction effects. On one hand, too short a time series length cannot be adequately captured by the TSP model. On the other hand, longer time series lengths mean more time is invested in the learning process and can also lead to harmful overfitting effects [40]. Tests show that our model can capture data trends and features most effectively when the sequence length *L* is set to 30. Our model was trained on a dataset constructed from a single evolution trajectory. We chose to obtain this trajectory under conditions of a weak system-environment coupling strength γ=0.5 and a long total evolution time T=40. Under these conditions, the system evolves slowly and has sufficient variations, providing ample material to construct a rich dataset. The reference time evolution trajectories were generated numerically by solving the Lindblad equation using the mesolve solver in the QuTiP 4.7.1 (Quantum Toolbox in Python) package [41]. We used these reference data to construct our dataset and the time series data for prediction. We sampled 240 times per unit time, obtaining a total of 7170 data points. We used 80% of the data for the training set and 20% for the test set. Subsequently, we extracted 30% of the data from the training set as the validation set to monitor the model’s training progress.

**Figure 2:**
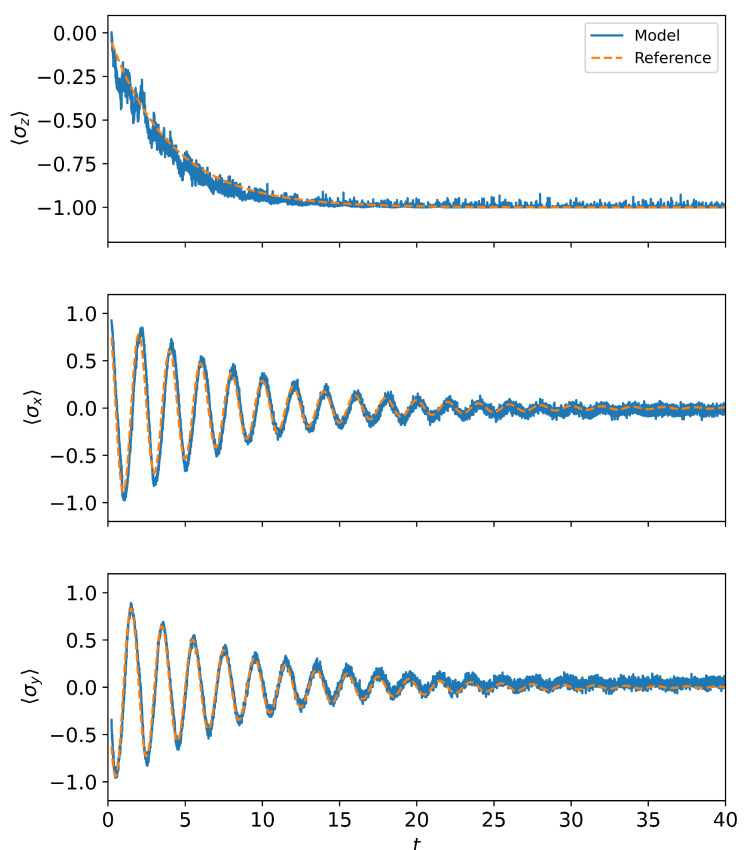
The expectation value $\langle \sigma_{z}\rangle$, $\langle \sigma_{x}\rangle$, and $\langle \sigma_{y}\rangle$ as a function of time *t*. The variable *t* represents the evolution time. The coupling intensity γ=0.5, g=−π/2, and the initial state is $|+\rangle \left(\langle \sigma_{x}\rangle =1\right)$.

**Figure 3:**
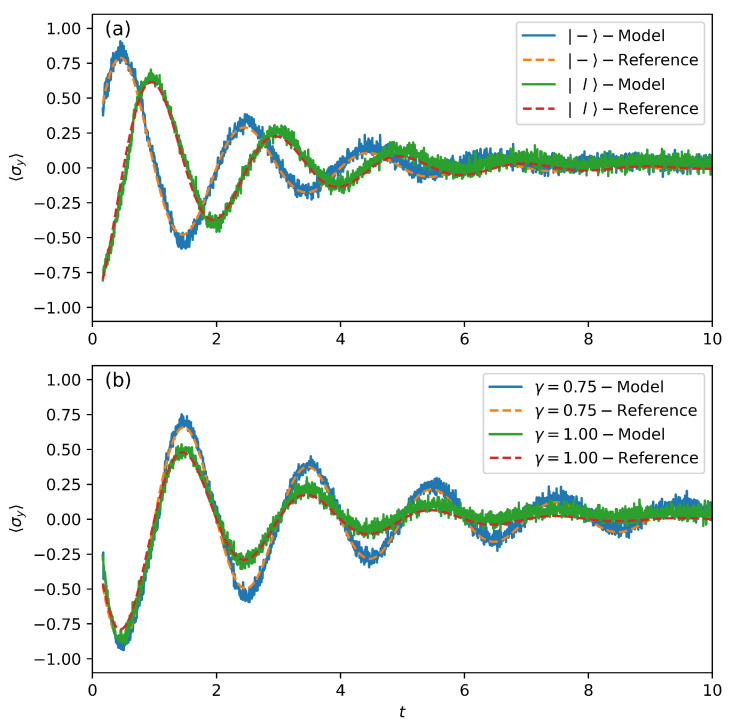
The expectation value $\langle \sigma_{y}\rangle$ as a function of time. (**a**) The different initial states are $|l\rangle \left(\langle \sigma_{y}\rangle =-1\right)$ and $|-\rangle \left(\langle \sigma_{x}\rangle =-1\right)$. The coupling intensity γ=1. (**b**) The different coupling intensities are γ=0.75 and γ=1. The initial state is $|+\rangle$.

In Figure 2, we present the predictions of the three Pauli operators’ expectation values $\langle \sigma_{z}\rangle$, $\langle \sigma_{x}\rangle$, and $\langle \sigma_{y}\rangle$ computed from the density matrix by the TSP model. The comparison with the numerically calculated results shows that the TSP model can accurately predict the oscillations or dissipations of the different Pauli operators’ expectation values for the open quantum system. It can also provide predictions that follow the trend of change when the average values continuously decrease and tend to a steady state in the long-time limit. We also test our model with different initial states and coupling strengths, as shown in Figure 3a,b. The results once again demonstrate that the trained model is capable of predicting the trends of dynamical evolution under different conditions. This indicates that our model has effectively learned the characteristics of the evolution and is capable of predicting future data based on past data. However, at the same time, the prediction results show more fluctuations and noise compared to the numerical reference results. Model uncertainty may be an important cause of the prediction noise. Specifically, for a given set of training data, there may exist multiple neural network models with similar performance but different internal parameters. The model obtained from a single training is just one random realization, and its predictions will carry the “individual noise” of that particular random initialization and optimization path. Lin et al. [42] showed that by constructing an ensemble of models and averaging the predictions of all models in the ensemble, the noise caused by the randomness of individual models can be significantly smoothed out, yielding a smoother trajectory that represents the average dynamical behavior.

### 4.2. Long-Term TSP

**Figure 4:**
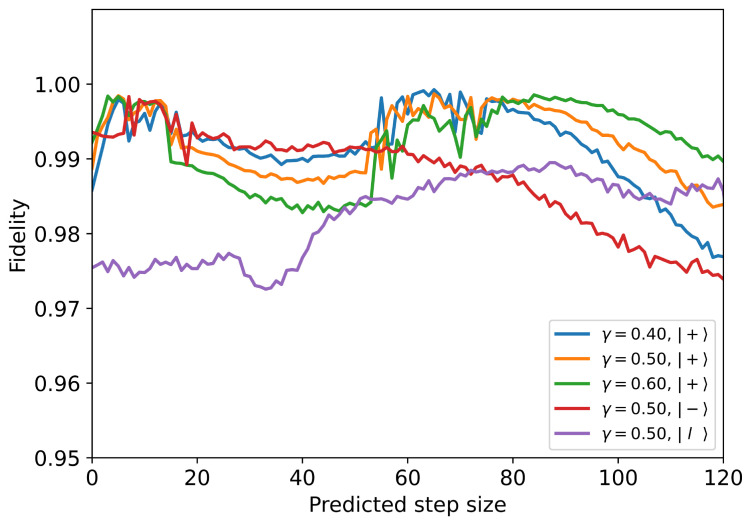
The fidelity of the density matrix for the long-term TSP under different initial states and different coupling intensities.

Next, we reorganize the time series using the predicted data, as depicted in Figure 1, to make further predictions for the future. When the prediction step exceeds the length of the time series, the predicted data completely replaces the original time series, and the model no longer relies on the original time series, that is, the long-term TSP. We select data from 30 time nodes starting from the initial moment to form our original time series. Using the long-term TSP strategy, we predict the reduced density matrix for the next 120 moments under different initial states and coupling intensities. The fidelity between the predicted density matrix and the reference density matrix is calculated by $F\left(\rho_{1},\rho_{2}\right)=\mathrm{Tr}\left(\sqrt{\sqrt{\rho_{1}}\;\rho_{2}\;\sqrt{\rho_{1}}}\right)$. Here, $\rho_{1,(2)}$ is the predicted (reference) density matrix, respectively. In Figure 4, we plot the fidelity versus the predicted step size for different initial states and coupling intensities for long-term prediction. From Figure 4, the fidelity exceeds 0.96 for all cases, indicating the accuracy prediction ability of our trained model. Note that the fidelity for different coupling intensities have their minimum value when the number of steps reaches approximately 40; the reason is that the reduced density matrix undergoes a significant change at this point. Small single-step prediction errors accumulate and amplify continuously in the autoregressive loop, eventually causing the prediction trajectory to deviate from a physically reasonable path, manifesting as non-physical oscillations, drifts, or divergences. As shown in our Figure 4, with the increase in prediction steps, the accumulated prediction error increases as expected, and the model’s prediction eventually diverges. The time required for the model to complete the prediction task is also an important metric for the TSP model. Therefore, we tested the time taken by the trained model to predict 120 time points and the time taken to generate a trajectory of 120 time points using QuTiP numerical integration, which were 0.167 s and 0.222 s, respectively. This indicates that, without considering the time cost of model training, our model has already demonstrated significant acceleration.

**Figure 5:**
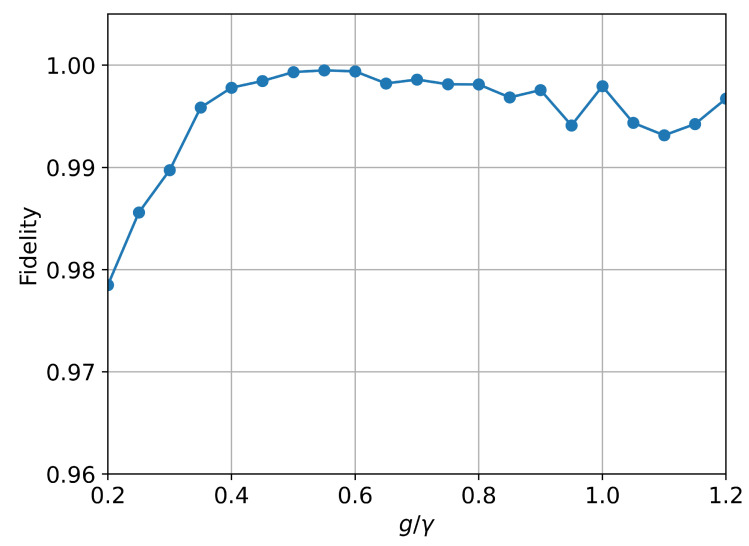
The expectation value $\langle \sigma_{x}\rangle$,$\langle \sigma_{y}\rangle$, and $\langle \sigma_{z}\rangle$ for an open quantum system to reach a steady state at different g/γ. The coupling intensity γ=0.5.

To test the generality of our approach, we now predict the steady state for the Hamiltonian $H=g\sigma_{x}$ and Lindblad operator $L=\sigma_{-}$. We choose to numerically calculate the trajectory under the conditions g=0.3, γ=0.5, and T=60 using QuTiP. We sample 80 times per unit time, obtaining a total of 4770 data points, and construct the dataset in the same proportion as the previous dataset. This physical system will reach a steady state after evolving for a certain period of time. We also use long-term TSP and select the 30 time points before reaching the steady state as the time series data to predict the steady state for different g/γ. Figure 5 shows the fidelity between the model’s predicted steady state and the numerical reference results for different g/γ when the system reaches the steady state. The fidelity between the model’s predictions and the reference values exceeds 0.975. This demonstrates that our training method can be applied to different systems and remains effective in long-term predictions.

## 5. Conclusions

This paper presents a deep learning model that integrates Transformer neural networks with TSP to simulate the dynamical evolution of open quantum systems. By adopting the POVM representation, we transform the system’s density matrix into a probability distribution, enabling the direct application of sequence-based learning frameworks. Numerical experiments demonstrate that the model achieves high-fidelity predictions of both short- and long-term dynamics under varied initial states and coupling strengths, and accurately captures the steady-state behavior of the system. Our work illustrates the potential of data-driven methods for simulating open quantum dynamics.

## Figures and Tables

**Figure A1:**
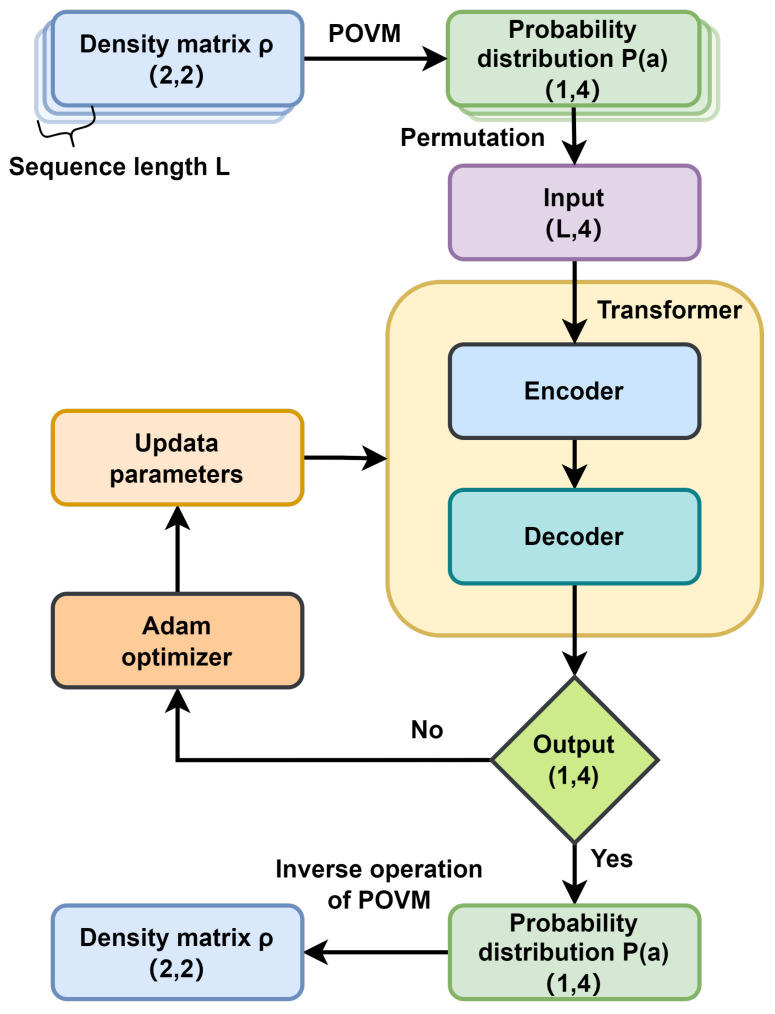
Schematic of the training process.

## Data Availability

The code implementing the models and analyses presented in this study is openly available in a GitHub repository at https://github.com/Wangzhaowei13/TSP_of_OQSD (accessed on 9 January 2026). The data are available on request from the corresponding author due to privacy or ethical restrictions.

## Abbreviations

The following abbreviations are used in this manuscript:

TSP	time series prediction
POVM	positive operator-valued measure
PINNs	physics-informed neural networks
QuTiP	Quantum Toolbox in Python

## Appendix A. Training Process and Hyperparameters of TSP Models

We plot the training process of the TSP model in Figure A1. Initially, the density matrices of the *L* consecutive time steps are transformed into probability distributions using the POVM method. Subsequently, these probability distributions are organized into an autocorrelated matrix to serve as the Input for the Transformer. Following the Encoder and Decoder layers, a predictive outcome, Output, is generated. This prediction is then assessed, and if it fails to meet the specified criteria, the Adam optimizer is utilized to fine-tune and update the Transformer’s parameters. Upon achieving the desired prediction, which indicates the completion of TSP training procedures, a trained TSP model is capable of offering predictive outcomes in the form of corresponding probability distributions. Finally, the predicted density matrix is obtained through the inverse operation of POVM.

The role of the Encoder in Transformer is to process the Input Matrix, converting it into a set of internal encoding that will capture the key information in the input sequence. The Encoder will utilize the self-attention mechanism and feedforward neural networks to extract and transform features of the input data to varying degrees. Subsequently, it will convert them into an internal encoding format and pass them to the Decoder.

The Decoder is the module used to generate the Output, which takes the output from the Encoder. The Decoder is also composed of multiple layers of self-attention mechanisms and fully connected layers. The internal encoding, after processing, will generate the corresponding output sequence. The details and functions of the Encoder and Decoder layers of the Transformer can be found in Ref. [32].

**Table A1:** Hyperparameters of TSP models.

Parameters	Model 1	Model 2
$d_{model}$	32	32
Feedforward network dimension	128	128
Number of attention heads	8	8
Positional encoding maximum length	5000	5000
Dropout rate	0.1	0.1
Learning Rate	$10^{-5}$	$10^{-5}$
Batch Size	20	20
Training Epochs	500	500
Optimizer	Adam	Adam
Weight initialization scheme	Default	Default
